# Co. Proceedings of N. Y. Pathological Society, in American Medical Monthly

**Published:** 1855-03

**Authors:** 


*Co. Proceedings of N. Y. Pathological Society, in American Medical Monthly.*—Dr. Dalton performed some experiments, showing the peculiar manner in which gastric juice, when present in a saccharine fluid, modifies the action of Trommer's test for grape sugar. When grape sugar or honey is dissolved in water, the addition of a drop or two of a solution of sulphate of copper, followed by an excess of liquor potassæ, produces a transparent blue solution, which on boiling, becomes yellow and opake, from the deposit of an abundance of the insoluble suboxide of copper. If the honey, however, be dissolved in gastric juice, the addition of sulphate of copper and caustic potash produces a solution of a purple color, instead of a blue, which in boiling changes to yellow, but at the same time remains perfectly clear, the suboxide of copper being apparently produced as usual, but being retained in solution instead of being deposited as a precipitate. Gastric juice has this effect though the honey be present in very large proportion. A mixture of the two in equal volumes, for example, does not precipitate the copper when tried immediately with Trommer's test; but if it be diluted beforehand with water, the yellow suboxide is deposited as in ordinary fluid. Though the copper is not usually precipitated by Trommer's test from gastric juice containing grape sugar, yet the change of color from purple to yellow, which takes place in boiling, is an equally good indication of sugar, when it is present in large or moderate quantity, say one-sixteenth of a drop of honey to the drachm of gastric juice, the purple color does not change in boiling, and the test is entirely interfered with.

Dr. Conant remarked, in continuation of the discussion on cholera, that the Mott street Hospital, of which he had charge, assisted by Drs. Stiles and O'Reilly, was opened on the 22d of July, and received the first patient on the morning of the 25th. There were in all, received in the Hospital, 329 patients, of whom 154 died, and 175 were discharged entirely free from cholera. During the epidemic Dr. C. made 104 post-mortem examinations, and found the appearances so similar in all, that he thinks they may be looked for with as much confidence as the pathological appearances in phthisis pulmonalis. What the choleraic influence is, he does not pretend to say, whether it be the cholera fly, bug, miasma, or contagion. He has seen no evidence of contagion, but is satisfied that the poison is engendered and perpetuated in proportion to the amount of material it has to work upon, in certain districts and localities. The diarrhœa, which patients are frequently troubled with two or three days previous to an attack of cholera, he thinks, is not produced by choleraic poison, but that it is rather accidental, to which all are more or less liable during the months in which cholera usually prevails.

He believes all are under the choleraic influence, but that it requires some depressing agent to bring the vital powers within its control, that this may be diarrhœa, or any other influence that acts directly to exhaust the nerve force. Hence, in many cases we need not have diarrhœa at all, only so far as to empty the intestines of the bilious matter they contain at the time of attack.

Dr. C. believes the choleraic poison acts directly upon the ganglionic system of nerves; that the functions of the sympathetic are paralyzed and for the time almost destroyed, so that no organ depending upon this system performs its functions regularly, and those most dependent, as the stomach and duodenum, least of all. The capillaries are deprived of their irritability and allowed to dilate; the fluids of the blood are thrown into the stomach and duodenum in fearful quantities, by a new process of transudation; as the stomach becomes full, it empties itself by the act of vomiting, &c. The watery portions of the blood are thrown out, the blood becomes thickened, the kidneys cease to act, perhaps for the want of serum to wash out the uriniferous tubes. The capillaries in the extremities are obstructed by the thick blood, reflex action manifests itself, and the muscles contract to assist the capillary circulation of the part. The general tendency of the blood is towards the stomach and duodenum, hence the collapsed state of the cutaneous capillaries.

Post-mortem examinations were generally made a few hours after death.

External appearances. Face contracted, eyes sunken, pupils slightly dilated, skin blue, especially the hands, face, and scrotum of the male.

Thorax. Pleura pulmonalis, found covered with a peculiar slimy substance, as though deposited by the absorption of fluids natural to the parts.

Hypostatic congestion usually well marked. Pericardium slightly moistened, upon the apex of the heart and upon the anterior portion of the lung, small patches of ecchymosis in some cases.

Abdomen. Peritoneum usually congested, except that portion covering the stomach and duodenum, where it was usually pale, always quite dry.

Spleen and pancreas generally quite natural.

Kidneys appear as if injected by mechanical force. Mucous membrane of the stomach has a peculiar par-boiled appearance, is quite soft and rolled up into large rugæ, as if by contraction of the muscular coat, with patches of congestion in most cases, but never having the appearance of inflammation. Mucous membrane of the œsophagus light-colored, extending half an inch into the stomach, where the line of demarcation was quite distinct. The pyloric orifice of the stomach usually almost closed by the large fold of mucous membrane. The valvular conniventes of the duodenum quite large. The mucous membrane of the jejunum slightly congested, with an increase of congestion at the ilio-cœcal valve; the mucous membrane of the colon, usually having no appearance of disease. The whole alimentary canalgenerally somewhat contracted.

The liver is of normal size and full of black blood, often presenting light spots on various parts. Gall bladder is filled with dark bile, except in two cases, when it was filled with a watery substance resembling bile only in taste. The urinary bladder is contracted to the size of a hen's egg.

Brain. The veins of the dura mater and arachnoid are found much distended with blood, with large quantities of serum in and around the brain. The ceregral arachnoid seemed in some cases to be œdematous, presenting a peculiar light, opake appearance. The vessels around the medulla oblongata filled with blood more red than in other parts of the brain. Upon dissecting the brain, the substance seemed to contain more blood than usual. No other pathological condition was found until he came to the pituitary body. Invariably this was found more or less hardened; in some cases so hard as to be with difficulty crushed between the thumb and finger. Now if the position be proved, that the pituitary body is the centre of all sympathetic action, Dr. C. thinks cholera is proved to be a disease of the sympathetic system, and *vice versa.*

Solly and some others are inclined to take this view of the function of the pituitary body. No pathological state was found of the abdominal sympathetic system.

Dr. C.'s treatment was the following:

If the patient was brought in before the bowels were entirely free from bilious matter, acetate of lead 3 grs. and opium 1 gr. was given every hour or two as the case might be. If this failed, or the patient was passing by stool, or vomiting rice-water when admitted, 3 grs. opium, 2 grs. acetate of lead, were given as the case demanded. External applications of mustard, and hot drinks were always used. With this treatment reaction was mild, and recovery was predicted with confidence, unless the patient had passed the stage of vomiting and purging. After this stage, calomel and rhubarb each grs. v. every three hours were given, with external appliances when required; then the hot drinks were particularly grateful, and Dr. C. has never heard a patient complain of the drinks being too hot, except when given luke warm, and after taking hot drinks they seldom called for cold. Beef tea and brandy were given by the mouth; but believing the mucus membrane of the stomach and duodenum were in a condition to throw out rather than to absorb, Dr. C. was in the habit of giving tonics and stimulants by injection, and in this way found they acted more powerfully. In cases where larger doses of laudanum seemed to produce no effect, one half the quantity by injection would apparently have its full effect, in a few minutes producing contraction of the pupil, &c.

If the patient was admitted moribund, very little medication was restored to. A careful inquiry was instituted in all cases, and it was found that more were attacked in the morning from one to three o'clock, and by looking over the deaths it will be found that more died from two to ten, P. M., than at any other hour. Dr. C. thinks that one week he was considerably under the choleraic influence himself, being waked about two o'clock every morning, for five in succession, by a peculiar sick feeling, which was averted by a dose of his own medicine.

(The tables of nativities and ages were added by Dr. Conant, for which see the Nov. No. of the Monthly. Ed.)

Dr. Hutchinson remarked that there was one remarkable post-mortem phenomenon peculiar to cholera, to which allusion had not been made, although it had been frequently observed; viz, the rise of animal heat in the body after death. He had in one case observed the temperature of the pelvic cavity one hundred and four degrees, three hours after death, whilst the temperature of the atmosphere was eighty-three degrees. In another case the temperature of the axilla was one hundred and four degrees, one hour after death, when the temperature of the atmosphere was eighty-seven degrees; and six hours after death, the pelvic cavity of the same subject gave a temperature of one hundred and seven degrees, atmosphere, eighty degrees. Dr. H. also mentioned another case, observed fourteen and a-half hours before death, in which the temperature of the mouth was seventy-eight degrees, side of the abdomen forty-nine degrees, calf of the leg eighty-seven degrees, vulva forty-nine degrees; and four hours after death the side of the chest gave a temperature of ninety-four degrees, atmosphere eighty-five degrees.

Dr. Hutchinson remarked that this phenomenon was not a novelty, it had often been noticed, but it was now brought up with the hope, if any gentleman present had investigated the cause, he would be able to offer us a rational explanation. Dr. H. stated he had frequently observed the blanched appearance of the external surface of the stomach and bowels of patients who had died of cholera, to which attention was directed at the last meeting by Dr. Clark in the plates of M. Perigoff. He had noticed the inflammatory appearance of the intestinal mucous membrane, but had ascribed it simply to a venous stasis of the blood, because the inflammatory effusions and other sequelæ of inflammation were absent, and in many cases also this injected condition of the capillaries was wanting. Dr. H. also, referred to an experiment made by Magendie, which consisted in the injection of an aqueous fluid into the intestinal arteries of cholera subjects, whose intestinal mucous membranes presented the reddened appearance alluded to, with the effect of bringing away the blood and leaving the intestine clear and white, which he thinks could not have been the case if the organic changes always resulting from inflammation had taken place.

